# Research on an Optimized Evaluation Method of the Bearing Capacity of Reinforced Concrete Beam Based on the Bayesian Theory

**DOI:** 10.3390/ma16062489

**Published:** 2023-03-21

**Authors:** Lifeng Wang, Ziwang Xiao, Fei Yu, Wei Li, Ning Fu

**Affiliations:** 1 School of Civil Engineering, Northeast Forestry University, Harbin 150040, China; 2 Heilongjiang Highway Survey and Design Institute, Harbin 150080, China; 3 China Railway 22nd Bureau Group Corporation Limited, Beijing 100043, China

**Keywords:** evaluation method, bearing capacity, reinforced concrete beam, Bayesian theory, MCMC sampling method, MH sampling method

## Abstract

An optimized evaluation method of the bearing capacity of reinforced concrete beam based on the Bayesian theory was proposed in this paper. This evaluation method optimized the traditional Markov Chain-Monte Carlo (MCMC) sampling method, and proposed an improved Metropolis–Hastings (MH) sampling method and a transitive MCMC (TMCMC) sampling method based on the MCMC theory. These two derived sampling methods solved the problem that the traditional MCMC algorithm makes it difficult to achieve convergence when the number of modified parameters is large. Therefore, on the basis of obtaining the measured sample information and the prior information of uncertain parameters, this paper first used multiple “model components” to form a model sample, then carried out a sensitivity analysis based on the relevant response indicators and selected the key parameters that had a great impact on the bearing capacity, carried out static load tests, and extracted and analyzed the experimental data. Then, based on a large amount of analysis data, the improved MH sampling method and TMCMC sampling method were used to establish a posterior probability distribution database. Finally, multiple posterior probability distributions were used to identify and predict the bearing capacity. The results showed that the method was feasible and effective for the evaluation of the bearing capacity of reinforced concrete beam.

## 1. Introduction

To strengthen the monitoring of the highway bridge system, comprehensively master its service status, and change rules have become the top priority to ensure the normal operation of highway traffic. Due to the large-scale operation of highway bridges, there is a need for performance maintenance in the whole life cycle. During the operation period of existing bridges, there will often be a certain degree of performance damage, causing its bearing capacity to continue to decline [1]. Especially in the context of the rapid development of the number and volume of infrastructure construction in the world, the requirements of bridge monitoring are also increasingly high. The bearing capacity and the expected life of the structure can be accurately determined, through the reliable monitoring and evaluation of the bearing capacity of the structure. Then, the corresponding theoretical support for the subsequent reinforcement and reconstruction of the structure can be obtained. 

Thus far, many scholars have studied the problem of bridge health monitoring and evaluation. Jin et al. [2,3] combined the vibration-based damage detection method with the artificial neural network based on the extended Kalman filter. They used the vibration acceleration and temperature data obtained from the bridge monitoring to identify and analyze the correlation between natural frequency and temperature. Then, they selected appropriate input variables for the neural network model to eliminate the influence of temperature. This method was used to assess the health of a 26-m long single span bridge in Meriden, Connecticut. Farreras Alcover et al. [4] proposed a fatigue reliability assessment method for welded joints of orthotropic bridge steel deck. This method used real data to characterize the pavement temperature and heavy traffic flux through the autoregressive model of pavement temperature and the autoregressive model combined with the traffic intensity regression model. They assessed the health of the Great Belt Bridge in Denmark by analyzing the field monitoring and measurement data. Sousa et al. [5] established the finite element model of the bridge using the assumed design material parameters and the creep function and shrinkage function calculated based on the long-term monitoring data. According to their model, a long-term health assessment of the Leziria Bridge in Portugal was carried out. Liu et al. [6] combined the virtual deformation method (VDM) with hyperelement technology, and proposed a model modification algorithm based on the hyperelement virtual deformation method for solid elements, which can quickly solve the global and local correction parameters of damaged bridges, and has been applied in real bridges. The research showed that this method can improve the calculation efficiency of model correction by reducing the number of structural degrees of freedom and avoiding the reconstruction of stiffness matrix on the premise of meeting the calculation accuracy, and was suitable for long-term refined health monitoring of complex damaged bridges. Sun et al. [7] discussed the application scenarios of big data and artificial intelligence in bridge health monitoring based on the massive data processing of structural health monitoring, and pointed out the applicability of deep learning algorithm in UAV detection and health monitoring. Kaloop et al. [8] studied the dynamic characteristics (dynamic acceleration, dynamic deflection, frequency, and damping ratio) of highway steel plate girder bridges using strain measurement methods, designed a transformation function based on polynomial prediction model and dual filtering method, and used cyclic filtering to eliminate real-time strain measurement noise in the time domain to predict the dynamic behavior of bridges. The research results show that only monitoring the structural dynamic strain can accurately predict the dynamic deflection and acceleration of the bridge in the short-term performance evaluation, so as to reduce the difficulty of short-term monitoring of steel plate girder bridges. Fradelos et al. [9] used approximate analysis image technology to collect the vibration signal of an arch bridge with a low-cost camera, and analyzed the natural frequency and damping ratio of the structure according to the dynamic deflection signal. The research showed that it was possible to reconstruct the two-dimensional kinematics of the flexible bridge by using the image taken by the non-precision camera, but the movement track of the control point is required to be clearly visible on the image, and there should be vertical and horizontal bridge elements defining the image scale beside each control point, which will undoubtedly increase the difficulty of bridge health monitoring and evaluation. Tan et al. [10] studied the modal parameter variability of highway cable-stayed bridges based on the vehicle-bridge coupling theory, adopted vehicles with accelerometers to pass through the measured bridge, extracted the modal vibration mode of the bridge to identify the local impact damage of the bridge and the overall foundation scouring damage, and gave the calculation method of bridge damage assessment. Wu et al. [11] superposed the rotation angle of the bridge end and the modal parameters of the bridge, analyzed the monitoring data, and verified the algorithm on a cable-stayed bridge, proving that the cumulative slip can be used to predict the damage and residual life of the bridge sliding bearing. Bao et al. [12] curvatured the modal data before and after the damage of the simply-supported beam and the continuous beam, combined with the damage index of the bridge structure, used the BP neural network to identify the location of the structural damage, so as to predict the degree of structural damage, and compared the results with the real value to achieve the purpose of health monitoring and evaluation. He et al. [13] used wavelet packet transform to filter and reconstruct the original structural response signal, including vehicle-bridge coupling vibration, and established a damage identification method based on convolution neural network and recursive graph. Compared with the traditional statistical pattern recognition method, the convolutional neural network can achieve more accurate damage location and damage degree identification through intelligent learning layer by layer. Tang et al. [14] established a fine solid element model and a macro beam element model for the junction area of the tower and beam of a rigid frame system cable-stayed bridge. Based on the natural vibration mode frequency and static load response angle of the solid element model, the objective function was constructed. The local stiffness parameters of the macro beam element model were optimized and modified using the artificial bee colony algorithm to achieve the purpose that the static and dynamic performance of the macro beam element model of the bridge can better approach the solid element model. Shabbir et al. [15] incorporated the genetic algorithm into the finite element model modification. According to the difference between the finite element model of the cable-stayed bridge and the modal parameters (natural frequency and vibration mode) provided by the sensor, the objective function of the genetic algorithm was defined. The health monitoring and evaluation of the bridge was carried out by comparing the updated finite element model with the measured frequency of the bridge. He et al. [16,17] calculated and predicted the long-term deflection growth coefficient of the structure through the three-stage bridge long-term deflection calculation method based on the long-term deflection observation results before and after the reinforcement of the Humen Bridge continuous rigid frame bridge and the effective prestress measurement data. Hou et al. [18] carried out the reliability evaluation of stay cables based on the results of monitoring the moving load, stay cable temperature, wind load, and stay cable force in one year. The research showed that the temperature, wind load, and moving load of the stay cable obey β, while the cable force follows the logarithmic normal distribution. Moreover, the two-stage performance equation was further used to evaluate the reliability of the cable. Based on the statistical principle, Mao et al. [19] used the model-free index training method in the damage early warning theory to evaluate the safety status of the Wanbu Bridge, and compared the real-time monitoring data of the monitoring system with the safety threshold to evaluate the safety status of the bridge. Xiao [20] analyzed the reliability of the bearing capacity of the bridge structure based on the health monitoring data for a period of time, and discussed the influence of the temperature effect on the reliability index of the bearing capacity of the structure by establishing the reliability evaluation method of the bearing capacity of the bridge based on the random vehicle load model. Chen [21] proposed a fatigue reliability analysis framework for long-span multi-load suspension bridges based on the health monitoring system and applied it to the Tsing Ma Bridge in Hong Kong. He defined the limit state function of fatigue reliability and established a probability model based on the monitoring data to determine the daily random stress response of multiple loads at the critical location of fatigue. After determining the probability distribution of the sum of the stress amplitude “m” power in a given time period, the health status of the bridge at different critical fatigue positions can be obtained. Chen et al. [22] evaluated the health status of concrete box girder bridges during normal operation based on bridge health monitoring data and reliability theory. They take the standard value of concrete tensile strength as the resistance and the vehicle load response and the vertical temperature gradient load response as the load effect, establish the serviceability limit state equation, use the empirical mode decomposition method to extract the temperature trend term of the strain response of the top and bottom plates of the box girder for probability density function fitting, and finally calculate the failure probability of the top and bottom plates of the mid-span section of the box girder under the most unfavorable combination to evaluate the bridge health. Yue et al. [23] evaluated and scored quantifiable bridge monitoring and inspection data with fuzzy reasoning method, and directly evaluated and scored non-quantifiable degree description according to expert system. After comprehensive analysis and calculation, the scores of bridge safety, durability, and serviceability of each section were obtained, and then the bridge health status was finally determined by the weighting method. 

Through the research of existing literature, it can be learned that when some scholars use the real bridge monitoring data to revise and proofread the model, in order to avoid the impact of existing damage on the structure, such research is often carried out in the new bridge monitoring stage. Moreover, the paper involves a relatively short monitoring period. Although the modified model has the possibility of damage early warning in theory, there is no damage judgment example with a large time span that can track the development of damage. In addition, the selection of the number and type of modified parameters is one of the most challenging aspects in the finite element model modification. It is necessary to select the optimal parameter set to make the model calculation results converge quickly and meet the requirements of calculation accuracy, so this work is often difficult to achieve in complex bridges.

In order to avoid the problems mentioned above in the research of bridge health monitoring and evaluation, this paper proposes a new method of dynamic evaluation of bridge bearing capacity based on Bayesian theory. Combining Bayesian theory with statistical knowledge can not only help to effectively grasp the characteristics of data, but also help people to fit models and estimate parameters in the form of probability distribution, and help people better deal with complex data. In this paper, Bayesian theory, finite element software ABAQUS, and data analysis software Matlab were organically combined. Relying on the existing data information, the automatic real-time correction between data and simulation analysis model can be realized, so as to form a dynamic correction and real-time evaluation system for the whole life cycle of the bridge. This system obtains the posterior probability distribution of the model to be identified through Bayesian theory, using the measured sample information and the prior information of uncertain parameters and using the Bayesian formula. On the basis of obtaining the posterior probability distribution, the improved MH sampling method and TMCMC sampling method are used to establish the posterior probability distribution database, and multiple posterior probability distributions were used to identify and predict the bearing capacity. 

## 2. Identification of the Bridge Bearing Capacity Based on the Bayesian Theory

### 2.1. Basic Framework of Bearing Capacity Identification

**Figure 1:**
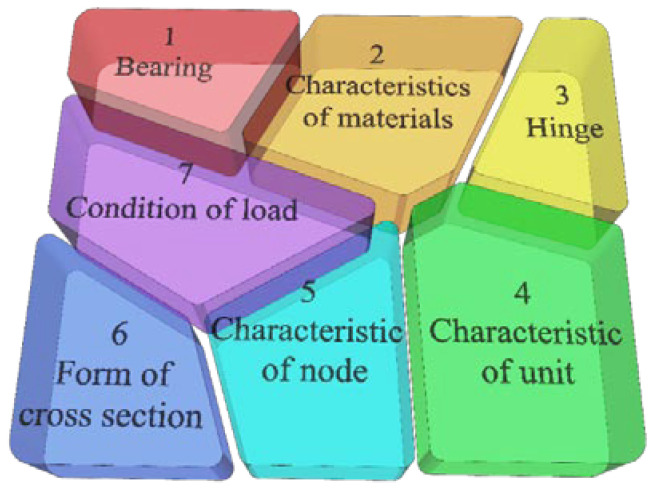
Schematic diagram of model components.

Generally speaking, structural uncertainty is mainly reflected in the expression of boundary conditions, section form, material heterogeneity, and analysis methods [24]. However, in this paper, the revised parameters of the finite element model will be automatically revised through the data iteration of the finite element software and the data analysis software. Therefore, there is no need to deal with the uncertainty of the analysis method separately. “Model components” is a specific element of uncertainty (in this paper, corresponding to the correction parameters, as shown in Figure 1). A series of “model components” can be combined into a complete model [25]. In the process of forming the model, the extraction of each component was independently redistributed, so the probability of the overall model can be obtained through the “model component” sampling probability.

The new method for evaluation of bridge bearing capacity based on Bayesian theory is to comprehensively analyze the cognitive error and random error of the structure, and combine each “model component” into a model sample. By comparing the test results and the prediction results of the finite element model, select the posterior probability distribution database that most conforms to the structural performance characteristics from the complex model samples to carry out structural response prediction and performance evaluation. This method is more scientific and accurate because it takes into account the influence of various uncertain factors [26].

The basic steps of bridge bearing capacity identification based on Bayesian theory are as follows:

(1) To observe the structure and obtain prior information. The distribution of uncertain parameters can be determined by analyzing the structure type, determining the material and load transfer path, and understanding the historical test data and other information.
(2) To establish models and select key parameters. Based on the known information, the finite element parent model was established. Sensitivity analysis was carried out using relevant response indicators. Additionally, key parameters that had a great impact on bearing capacity were selected.
(3) Structural testing. To carry out the test with corresponding test instruments to complete the collection of static data.
(4) Data analysis. By analyzing the static test data of the structure, the static test information of the structure was extracted.
(5) A posteriori probability distribution database and model identification were established by sampling. Based on the obtained analysis data and Bayesian theory, a representative posteriori probability distribution database was selected by the efficient sampling method to complete the identification of key parameters.
(6) Prediction and evaluation of bearing capacity. After establishing a posteriori probability distribution database, the bearing capacity prediction and performance evaluation were completed, by using multiple posteriori probability distributions in the database to meet the requirements of structural characteristics.

### 2.2. The Bayesian Formula and Derivation

The Bayesian formula is a famous formula of probability theory, which was proposed by Thomas Bayes, a famous British scholar. Bayesian analysis method combined sample information and prior information, which can make the analysis more accurate and faster. The core of Bayesian statistical theory is the Bayesian formula, which can solve the problems that are difficult to overcome by classical statistical methods. Given a system, remember that two events are A and B, then the Bayesian formula can be expressed as Formula (1) [27]:

(1) $$P(A/B)=\frac{P(A,B)}{P(B)}=\frac{P(B/A)P(A)}{P(B)}\propto P(B/A)P(A)$$

where P(A/B) is called the posterior probability of event A, P(A,B) is the prior probability of both events A and B, P(B/A) is called the likelihood function (conditional probability), P(A) is the prior probability of event A, and P(B) is called the marginal probability of event B. From Formula (1), it can be seen that the independent variable of P(A/B) is P(A) and is not related to P(B).

In classical statistics, the posterior probability of parameter θ is usually expressed as [28]:

(2) $$p(\theta /D)=\frac{p(D/\theta )p(\theta )}{\int_{\theta }p(D/\theta )p(\theta )d\theta }\propto p(D/\theta )p(\theta )$$

where P(θ/D) is the posterior probability of parameter θ, p(θ) is the prior distribution of the parameter θ to be repaired, D is the output response of model simulation, and p(D/θ) is the conditional probability distribution under the given θ, usually called the likelihood function.

Next, the Bayesian formula was introduced into the evaluation of bridge bearing capacity. In the given random system model group M, the output response of finite element software simulation is D, the prior probability of $M_{i}$ of the *i* th model, and the conditional distribution of the simulation results are $p(\theta /M_{i})$ and $p(M/M_{i},\theta )$, respectively. Then, the posterior probability of the modified parameter vector θ (($\theta =\delta_{1},\ldots ,\delta_{n}$), *n* is the number of modified parameters) corresponding to $M_{i}$ is [29]:

(3) $$p(\theta /D,M_{i})=\frac{p(\theta /M_{i})p(D/M_{i},\theta )}{\int p(\theta /M_{i})p(D/M_{i},\theta )d\theta }=\frac{p(\theta /M_{i})p(D/M_{i},\theta )}{p(D/M_{i})}\propto p(\theta /M_{i})p(D/M_{i},\theta )$$

where $P(\theta /D,M_{i})$ is the posterior probability of the modified parameter vector θ corresponding to $M_{i}$, $P(D/M_{i},\theta )$ is the posterior probability of the output response D corresponding to the modified parameter vector θ, $p(\theta /M_{i})$ is the prior probability of the *i* th model $M_{i}$, and the priori probability is $p(\theta /M_{i})=\prod_{i=1}^{n}p(\delta_{i}/M_{i})$. The relationship between the simulated value of the structure and the predicted value of the model is linear regression. Each probability distribution is Gaussian normal distribution. Since each system model $M_{i}$ is independent of each other, the modes of each order were independent and uncorrelated. For the system model group M, the posterior probability of θ can be simplified by (3):

(4) $$p(\theta /D,M)=\sum_{i=1}^{n}p(\theta /D,M_{i})p(M_{i}/D,M)=c\cdot \exp(-\frac{1}{2}J(\theta ))$$

(5) $$J(\theta )=\sum_{i}\sum_{j}(f_{i,j}^{E}-f_{i,j}^{M}(\theta ){)}^{2}/\sigma_{i,j}^{2}+{\left(1-MAC{\left(\theta \right)}_{i,j}\right)}^{2}/\sigma_{i,j}^{2}$$

where *c* is the constant factor of definite integral independent of θ, superscript E and M represent the modal test value and model prediction value, respectively, *f* is the modal frequency, $\sigma_{i,j}$ is the standard deviation of the *j* th mode of the *i* th model, and MAC is the modal confidence factor, representing the matching degree between the finite element mode and the working mode (as shown in Formula (6)): the closer the *MAC* value is to 1, the greater the correlation coefficient of the two vibration mode vectors.

(6) $$MAC_{i,j}=\frac{{\left[{\left(\varphi {\left(\theta \right)}_{j}^{M}\right)}^{T}\varphi_{j}^{E}\right]}^{2}}{\left[{\left(\varphi {\left(\theta \right)}_{j}^{M}\right)}^{T}\varphi {\left(\theta \right)}_{j}^{M}][{\left(\varphi_{j}^{E}\right)}^{T}\varphi_{j}^{E})\right]}$$

where $\varphi {\left(\theta \right)}_{j}^{M}$ and $\varphi_{j}^{E}$ represent the mode vector and standard mode vector predicted by the *j* th order model, respectively.

According to the maximum posteriori criterion, the optimal θ should be found according to the posteriori probability function of the parameter vector θ to be repaired, so as to maximize P(θ/D,M). According to the nature of the exponential function, the maximum value of Formula (5) was to be solved. The definite integral constant factor c in Formula (4) is related to the size of the posterior distribution. When the prior probability is a non-conjugate mixed distribution, or the dimension of the modified parameter vector is high (resulting in the exponential increase of the integral space), the calculation amount is too large to be solved by numerical integration or normal approximation. Therefore, based on the continuous development of statistical theory and technology, mathematical sampling methods are often used in engineering to replace and approximate the posterior distribution of parameters.

### 2.3. MCMC Sampling Method

MCMC sampling is an advanced and intelligent sampling method. The distribution of Markov chain samples obtained by MCMC is approximately equal to the posterior probability distribution of θ, and the expected variance of the modified parameters can be estimated according to the theorem of large numbers. MCMC has many types of sampling methods, of which MH method is the most commonly used [30,31]:

(1) Select the starting sample $\theta_{0}$ of the Markov chain with physical significance, so that its initial probability distribution $p(\theta_{0})>0$.
(2) The next sample in the Markov chain only depends on the current sample, and has nothing to do with other historical samples. Therefore, the proposed distribution $q(\theta /\theta_{i-1})$ is used to generate candidate sample $\theta_{c}$ based on the previous sample $\theta_{i-1}$.
(3) Based on candidate sample $\theta_{c}$ and candidate system model $M_{c}$, the acceptance probability function is calculated according to Formula (7):
  (7) $$\alpha (\theta_{i-1},\theta_{c})=\min(1,\frac{p(\theta_{c}/D,M_{C})q(\theta_{c}/\theta_{i-1})}{p(\theta_{i-1}/D,M_{i-1})q(\theta_{i-1}/\theta_{c})})$$
  where $\alpha (\theta_{i-1},\theta_{c})$ is the sample acceptance probability, $p(\theta_{c}/D,M_{C})$ is the posterior probability of candidate sample $\theta_{c}$ corresponding to $M_{c}$, and $q(\theta_{c}/\theta_{i-1})$ is the probability of generating candidate sample $\theta_{c}$ based on the previous sample $\theta_{i-1}$.
(4) Generate random number *μ* in (0,1) uniform distribution. When $\alpha (\theta_{i-1},\theta_{c})\geq \mu$, accept candidate point $\theta_{c}$, and then iterate again from step (2). On the contrary, reject candidate point $\theta_{c}$ and take $\theta_{i}=\theta_{i-1}$.
(5) Repeat steps (2)–(4) to finally produce a convergent Markov chain: $\{\theta_{0},\theta_{1},\cdot \cdot \cdot ,\theta_{i}\}$, which represents the candidate sample model.

When the number of modified parameters is large, the traditional MCMC algorithm is difficult to reach convergence due to the increase of rejection rate of new samples. Moreover, MCMC sampling is often “trapped” in a place with high local probability. Therefore, it is necessary to improve the traditional MCMC method.

### 2.4. Improved MH Sampling Method

In the improved MH sampling method, the concept of the window function was introduced to solve the problem of defects in the traditional MCMC method mentioned in the previous section. The optimization innovation of this method was to avoid direct sampling from the complex objective function, and to establish a posteriori probability distribution database through continuous sampling in two stages. The process was divided into two stages.

In the first stage, as described in the previous section, a priori distribution was used to generate random numbers. Moreover, the MH method was used to screen samples. The core of the second stage was to use the candidate samples in the previous stage to generate new samples, so as to reduce the rejection probability of sampling. The specific process is described below.

The candidate samples generated in the previous stage were used as the initial samples. The window function was calculated according to the initial sample. The window with a small probability value was enlarged, while the window with large probability value was reduced [32]:

(8) $$\lambda =\prod_{i=1}^{N}(\theta_{i}{)}^{\frac{1}{i}}/{\left(\theta_{i}\right)}^{\frac{1}{V}}$$

where λ is the probability value of window function and $\theta_{i}$ is the original parameter quantity of the *i* th sample.

The window function dimension parameters are shown in Formula (9):

(9) $$\omega ={\left(\frac{4}{N\cdot (V+2)}\right)}^{\frac{1}{V+4}}$$

where ω is the window function dimension parameter, *N* is the number of samples, and *V* is the dimension size of the sample.

We can use Equations (8) and (9) to calculate the probability density kernel value:

(10) $$\upsilon (\theta )=\frac{1}{{\left(\omega \cdot \lambda \right)}^{V}}$$

where υ is the probability density kernel value.

The candidate probability cumulative distribution function was constructed from each sample and the normalized probability density kernel value, the covariance matrix was calculated. Moreover, a new sample was generated from the previous sample. We needed to determine whether the new sample was accepted according to the Metropolis criterion, and obtain the Markov chain through iteration. Finally, the final posterior probability distribution library was formed by removing the sample model affected by the initial value.

### 2.5. Sampling Method of TMCMC

The above improved MH sampling method can be applied to the probability density distribution function with “peak and valley”. However, because the window function needs to estimate the window function of each intermediate probability density distribution function, if the number of parameters is too large, the sampling method will slow down. The TMCMC method adopts the idea of selecting a series of intermediate probability density distribution functions to converge to the objective function in stages. Its advantage is that TMCMC can select the intermediate probability density distribution functions by itself, which can better solve the problem of high-dimensional parameters. It is more applicable to the actual structural analysis. Moreover, in TMCMC, the probability density distribution function of the next stage was obtained by resampling on the basis of the probability density distribution function of the current stage, which can ensure that the Markov chain will not be “trapped locally” and solve the defect problem of traditional MCMC. The basic steps of the TMCMC sampling method were as follows [33,34]:

(1) Select a priori distribution, j=0, $f_{1}(x)=f(x/M)$, and sample it to obtain $\{x_{l,j}:l=1,\cdot \cdot \cdot ,N_{0}\}$, j=0,1,⋅⋅⋅,m.
(2) Calculate the variance of probability density weight $\omega_{l,j}=f{\left(D/x_{l,j},M\right)}^{p_{j+1}-p_{j}}$, $l=1,\cdot \cdot \cdot ,N_{j}$ to make $\sigma_{\omega }$ close to the preset threshold value to determine $p_{j+1}$.
(3) The initial value $x_{k,j+1}$ is obtained by resampling and the Markov chain is constructed. The proposed distribution is a normal distribution with $x_{k,j+1}$ as the mean and $\sigma_{j}$ as the variance. Conduct MCMC sampling, and use the Metropolis criteria [35] to judge whether to accept it, obtaining $f_{j+1}(x)$, $\{x_{l,j+1}:l=1,\cdot \cdot \cdot ,N_{j+1}\}$ in the next stage, at this time j=j+1.
(4) Repeat step (2) until j=m. After *m* stages, $f_{m}(x)=f(x/D,M)$, sample $\{x_{l,m}:l=1,\cdot \cdot \cdot ,N_{m}\}$ formed an approximate posterior distribution function.

### 2.6. Response Prediction of Bearing Capacity

After the posterior probability distribution database was established, the posterior probability distribution database can be used to predict the response of bearing capacity. Assuming that the response to be predicted is R and the predicted response value of the *i* th posterior probability distribution in the database is $R_{i}$, the probability distribution of bearing capacity response prediction is [36]:

(11) $$P(R/D,H)=\sum_{i}P(R_{i}/M{\left(\theta \right)}_{i},H_{i})P(M{\left(\theta \right)}_{i}/D)$$

where D is the output response of software simulation, $H_{i}$ is the excitation applied (such as various static and dynamic loads), $M{\left(\theta \right)}_{i}$ is a parameter θ of the *i* th posterior probability distribution in the corresponding posterior probability distribution database, $P(R_{i}/M{\left(\theta \right)}_{i},H_{i})$ is the predicted response corresponding to the *i* th posterior probability distribution under the influence of the excitation, and $P(M{\left(\theta \right)}_{i}/D)$ is the predicted response under the influence of the *i* th posterior probability distribution.

## 3. Verification Example: Evaluation of the Bearing Capacity of the Test Beam

**Figure 2:**
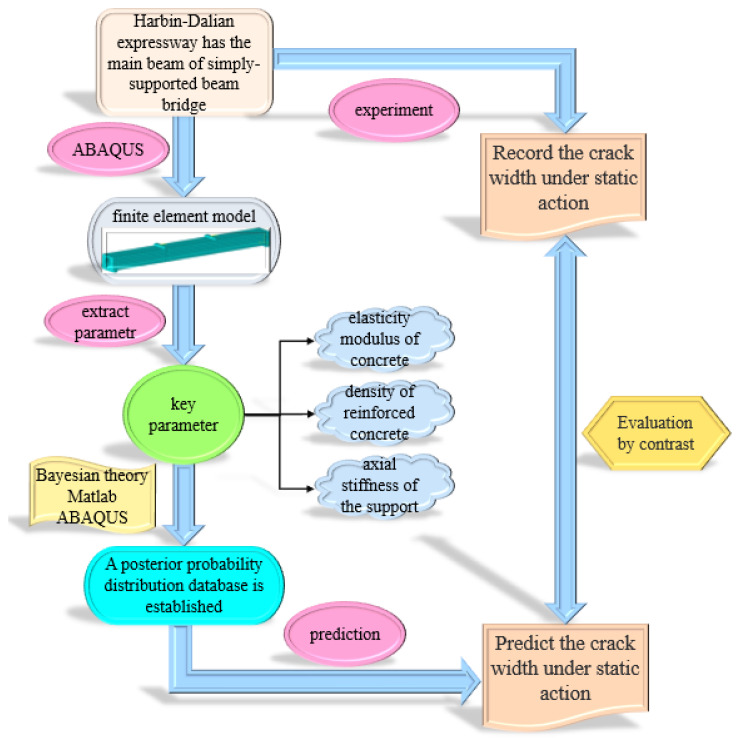
Flowchart of evaluation of the bearing capacity of the test beam.

To verify the feasibility and effectiveness of the new method for the evaluation of the bearing capacity of test beam bridges based on the Bayesian theory. In this paper, the experimental results of the main beam failure of the test beam bridge were strictly compared with the data obtained by the new method of evaluation of the bearing capacity of the test beam bridge based on the Bayesian theory. Additionally, the feasibility and effectiveness of the new method will be illustrated by the comparison results. Moreover, ABAQUS finite element software was used for modeling, and the programming function of Matlab was used for parameter assignment iteration and parameter analysis to realize sampling based on Bayesian theory. For the joint simulation of ABAQUS and Matlab. Firstly, we complete the finite element modeling in ABAQUS and obtain the automatically generated .inp file after the simulation. Secondly, we modify the .inp file according to the required parameters and then set req.txt according to the results to be read. Thirdly, we use Matlab to call the engine interface of ABAQUS and submit the .inp file to run and get the .odb file. Fourthly, we run the python script through Matlab, read the simulation result data from the .odb file, and save it to the resulting .txt file according to the requirements of req.txt. Finally, we use Matlab to read the data in the resulting .txt file. Based on the actual stress state of reinforced concrete test beam structure, three model components with high uncertainty (such as concrete elastic modulus, concrete density, and bearing stiffness) were selected to establish the finite element model. The specific evaluation process of the bearing capacity of the test beam is shown in Figure 2.

### 3.1. Destructive Test of the Bearing Capacity of the Test Beam

#### 3.1.1. Project Overview

**Figure 3:**
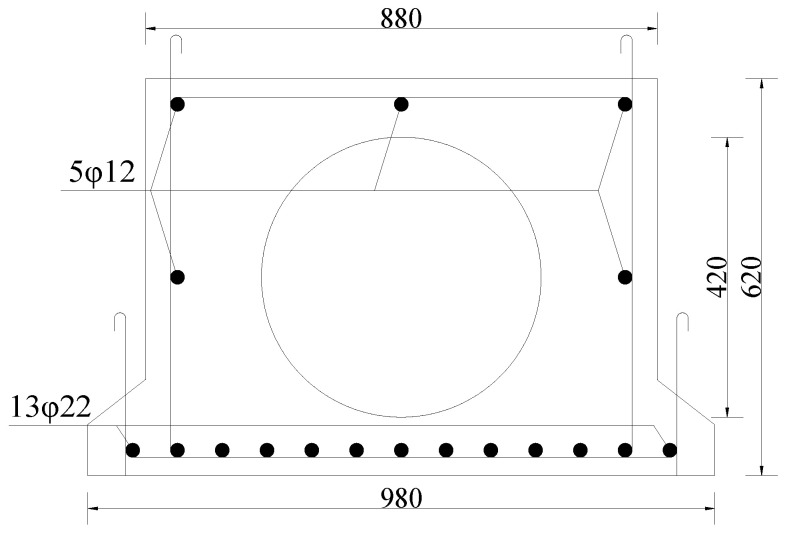
Cross-section of the main beam (unit: mm).

**Figure 4:**
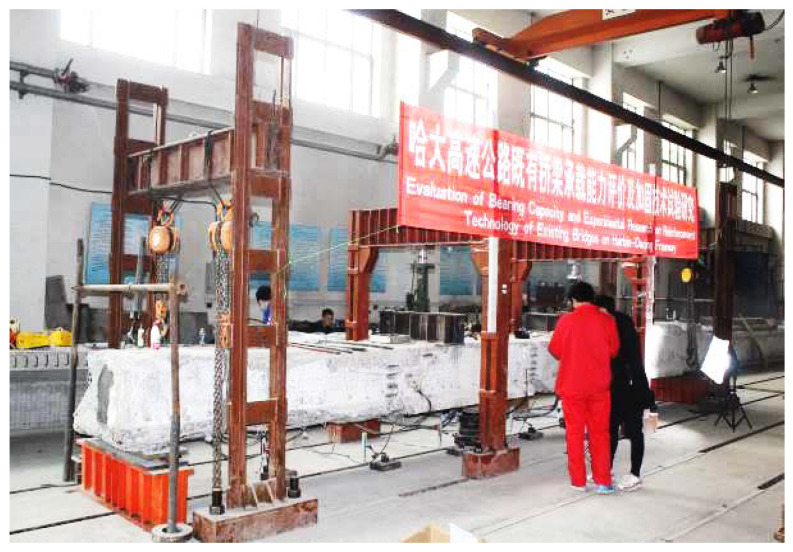
Experimental site.

Based on the reinforced concrete main beam of the existing simply supported bridge of the Harbin–Daqing Expressway. A test beam with a total length of 9.96 m was selected in this paper. Moreover, 13 φ22(HRB335) steel bars were equipped at the bottom of the beam, and φ8(HPB235) was used for the stirrups. The spacing of stirrups at the mid-span position was 200 mm and the spacing of stirrups at the fulcrum position was reduced to 150 mm and 75 mm. The concrete grade was C40, and the compressive strength was 40 MPa. The preparation of 1 cubic meter of this concrete requires 432 kg of cement, 168 kg of water, 558 kg of sand, and 1242 kg of crushed stone. The cross-section structure of the slab beam is shown in Figure 3, and the experimental site is shown in Figure 4.

#### 3.1.2. Support Device of Plate Beam

In order to make the stress of the test beam closer to the actual stress situation, the test beam support adopts the special simply supported hinge support and vertical support.

#### 3.1.3. Loading Position of the Plate Beam

**Figure 5:**
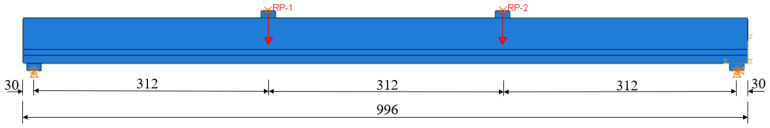
Schematic diagram of the plate beam loading (unit: cm).

This destructive test of bearing capacity adopts jack loading. In order to make the overall force of the beam uniform, the load action center applied by the jack to the beam is located at a distance of 312 cm from the two supports. The specific loading position is shown in Figure 5.

#### 3.1.4. Plate Beam Experimental Loading Device

**Figure 6:**
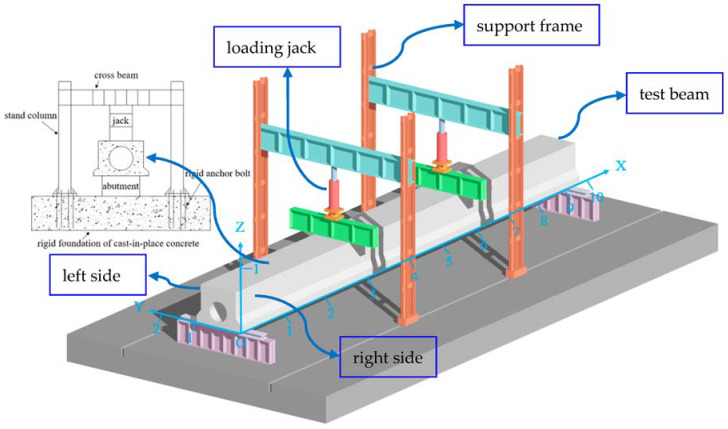
Schematic diagram of the loading device (Unit: m).

The failure load and reserved safety factor of the test beam shall be fully considered, and the design load of reaction frame and foundation shall be considered as 1000 kN. The load is applied through the reaction frame and jack, and the pressure sensor in contact with the jack is used to control the applied force. The corresponding loading device is shown in Figure 6.

#### 3.1.5. Experimental Loading Scheme Design

The experimental loading design of the main beam is shown in Table 1.

#### 3.1.6. Experimental Loading Steps

The specific loading steps are as follows:

(1) Before the formal test, the test beam shall be preloaded first. The calculated bearing capacity (Fu) with a preload grade of 0.05 times was applied twice to check whether the test instrument and sensor work normally, to ensure that the test beam and support and jack were in close contact, and whether the load was eccentric, so as to reduce the test error. After the check was correct, we zeroed the instrument equipment and entered the formal test stage. Note that the preload should control the force within the elastic range and should not produce cracks and other forms of loading residual value.
(2) When the load reached 90% of the technical value of the load for the bearing capacity test. The load shall be 0.05 Fr per level until the structure reaches the limit bearing capacity mark specified in 7.3.3 of “The Standard for Test Methods of Concrete Structures (GB/T 50152-2012) [37]”. That means that the concrete in the bending and compression area of the flexural member is subject to compression cracking and damage.
(3) We continued to load (the loading amount was controlled by deformation) until the structure was completely destroyed; during the loading process at this stage, the structure will be completely damaged when loaded at 0.5 kN/s.

#### 3.1.7. Crack Development of Main Beam

The crack width was measured and the extension rule of the crack was drawn through the crack width gauge and crack card. A meter ruler was used to measure the crack spacing and to draw the crack development map.

**Figure 7:**
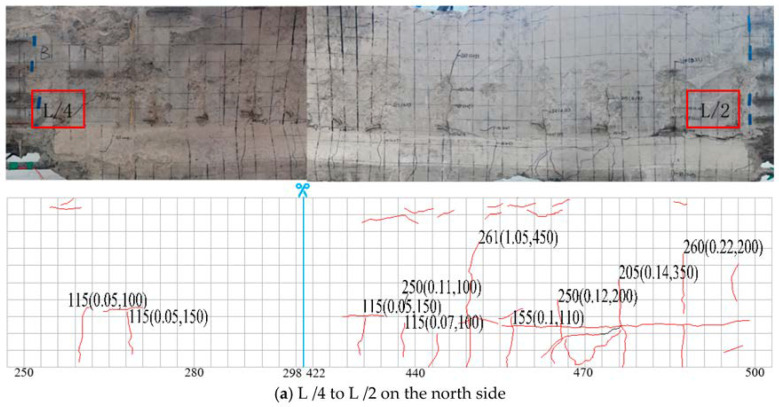

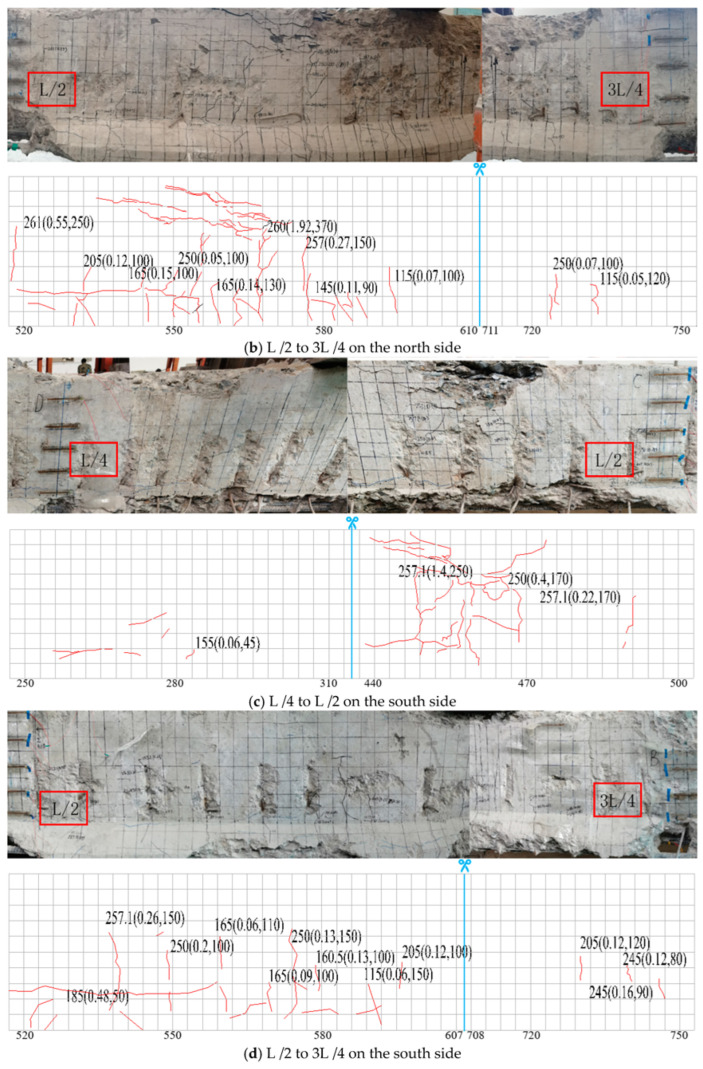
Photo and description of the main beam crack.

The distribution position, width, and development speed of cracks are the direct elements to characterize the performance of components, and are also the most intuitive and easily observed basis for judging the service performance of components [38]. The appearance and development of cracks affect the integrity and stress performance of components. The possible failure mode of components can be determined according to the type and distribution of cracks. The rapid development of cracks indicates that the components are about to reach the limit state. Therefore, it is necessary to observe and analyze the development of cracks during the stress process of components. A photo and description of the cracks in the test beam during loading are shown in Figure 7.

The meaning of the number combination is explained in Figure 7. Taking the figure “165 (0.09, 100)” as an example, this means that the pressure on the beam body when the crack occurs here is 165 kN, and the width and development height of the crack were 0.09 mm and 100 mm, respectively; the scale value unit in the drawing is cm.

According to the observation results of this loading test, during the loading process of the test beam, the crack development process is roughly divided into the following three stages:

(1) The crack develops slowly at the initial stage;
(2) Inclined cracks appear;
(3) The crack flourished until it was destroyed.

**Figure 8:**
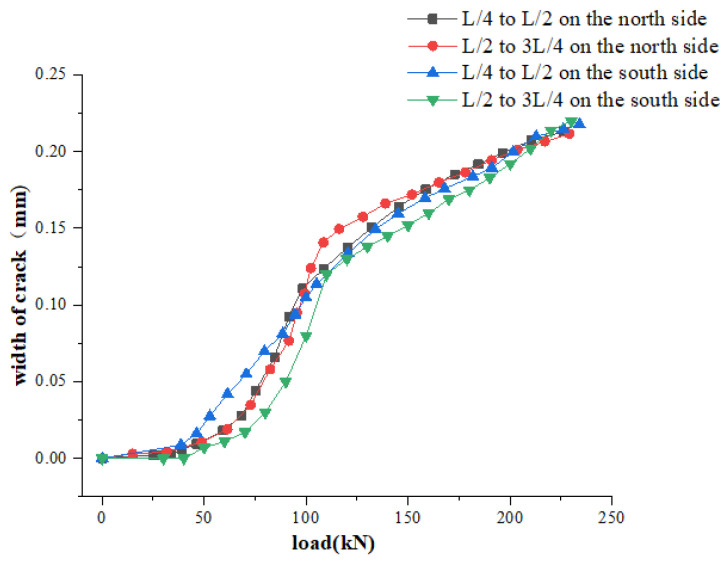
Change curve of the crack width of the main beam.

When the loading value was 65 kN, there were visible cracks near the midspan of the main beam. With the increase of the load, the small cracks that have been produced continue to extend upward, and the width continues to increase. When loaded to 180 kN, the main tensile stress in the flexural shear zone exceeds the tensile strength of the concrete, and oblique cracks appear. When the loading value was 232 kN, the maximum crack width was 0.22 mm, which exceeds 0.20 mm, reaching the crack width limit of the normal service limit state, local concrete was broken, and the main beam was damaged. The change curve of the main beam crack width is shown in Figure 8.

### 3.2. Establishment of Finite Element Parent Model

This section relies on the ordinary reinforced concrete main beam of the existing simply supported bridge of the Harbin–Daqing Expressway, and uses ABAQUS finite element software to establish the finite element main beam model.

#### 3.2.1. Sensitivity Analysis of Key Parameters

**Figure 9:**
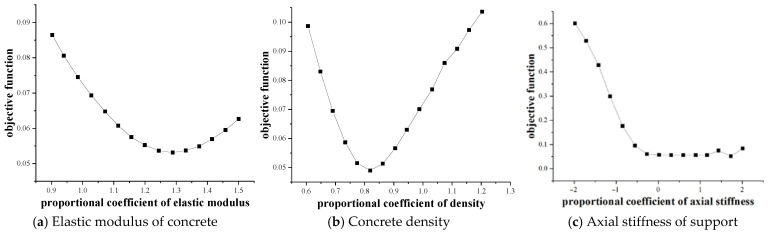
Sensitivity analysis of model components.

First, a sensitivity analysis was conducted to study the sensitivity of model components to the structural response, determine the reasonable physical parameters and value range in the structural model, and carry out sensitivity analysis on the three model components of the reinforced concrete continuous beam. The value range of model parameters is shown in Table 2 [39]. The specification value of C40 concrete elastic modulus in the table is *E*_0_ = 3.25 × 10^4^ MPa, the density of reinforced concrete was taken as *ρ*_0_ = 2450 kg/m^3^, and the axial stiffness of the bearing is taken as *K*_0_ = 100 kN/mm with reference to the test identification value of the same type of test beam. At the same time, proportional spacing is the general term for fast Fourier transform analysis of data. The linear average analysis method was adopted for the proportional spacing of elastic modulus and density, which means that each given data block is subject to fast Fourier transform one by one, and then the spectral value of each frequency point was subject to equal weight linear average. The logarithmic average analysis method is adopted for the proportional spacing of the support stiffness, which means that the given data block is subject to FFT. The result of the last FFT spectral value analysis accounts for half of the weight of the final average FFT spectral value, while the average FFT spectral value of all other analyses accounted for the other half of the weight. The objective function of sensitivity analysis was usually defined as the relative difference between the predicted value and the test value. The objective function defined in this section is based on the first *n*-order frequency and vibration mode of the test beam, as shown in Formula (12) [40]:

(12) $$obj(x)=\frac{\sum_{i=1}^{n}(|\frac{f_{E}-f_{A}}{f_{E}}|+1-MAC)}{n}$$

where obj(x) is the objective function, x is the model parameter to be identified, f is the natural vibration frequency, MAC is the modal confidence factor, and subscripts *E* and *A* are the test value and ABAQUS prediction value, respectively. The sensitivity analysis of model components first establishes the finite element model of reinforced concrete test beam in ABAQUS, assigns different model parameter values and assigns them to the model in ABAQUS through Matlab software, and obtains the objective function values of different models using Matlab and ABAQUS. Figure 9 shows the sensitivity analysis results of model components based on modal parameters. The research results show that the three model components are sensitive to the modal parameters of the reinforced concrete test beam and there is a theoretical optimal value. The subsequent research in this paper is based on the above three model components and their reasonable value range to establish the finite element model of the reinforced concrete continuous beam.

#### 3.2.2. Concrete Parameter Setting in ABAQUS

Concrete is a heterogeneous material, for which ABAQUS provides two constitutive models, namely the plastic damage model and mixed dispersion cracking model [41]. The plastic damage model is not only suitable for static and dynamic analysis, but can also better simulate the stress, deformation, and higher convergence of the structure [42]. Adopting reasonable plastic damage model parameters are the key step of concrete numerical analysis. These parameters can accurately capture the mechanical behavior of concrete. The ABAQUS user manual describes the expansion angle *ψ*, eccentricity e, ultimate strength ratio of biaxial bearing to uniaxial bearing *f*_b0_/*f*_c0_, ratio of second stress invariants *K*, and viscosity coefficient μ. The physical meaning of the parameters and the reference values were provided. Liu [43] used the parameters in Table 3 to establish a reinforced concrete beam model. The simulation results of these parameters are in good agreement with the experimental results. This paper also uses the values of these five key parameters.

ABAQUS uses the plastic damage model for analysis. The stress–strain relationship needs to be converted into a different stress–strain relationship. The inelastic strain is calculated by Formulas (13) and (14):

(13) $$\varepsilon_{c}^{in}=\varepsilon -\sigma_{c}/E_{c}$$

(14) $$\varepsilon_{t}^{in}=\varepsilon -\sigma_{t}/E_{c}$$

where $\varepsilon_{c}^{in}$ and $\varepsilon_{t}^{in}$ are the concrete compression inelastic strain and compression inelastic strain, respectively, $\sigma_{c}$ and $\sigma_{t}$ are the compressive stress and tensile stress, respectively, and $E_{c}$ is the elastic modulus of concrete.

**Figure 10:**
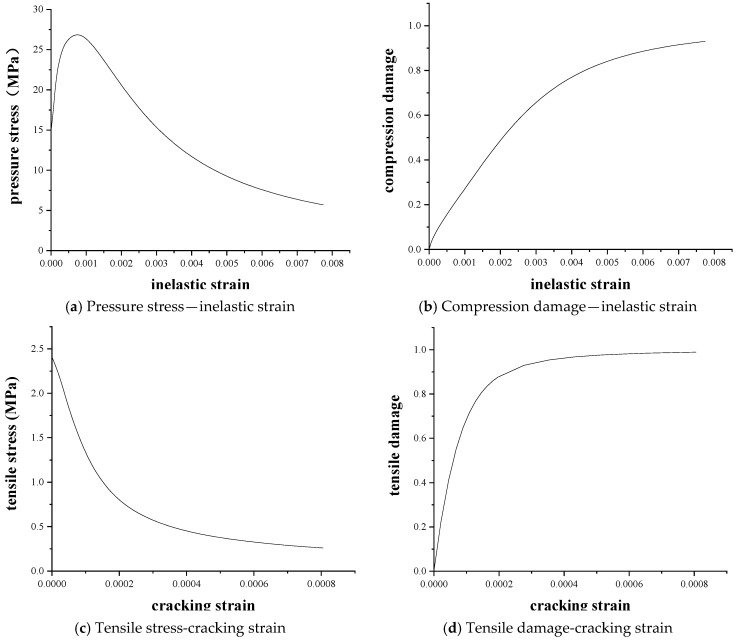
Constitutive relation curve of concrete.

The mechanical properties of concrete and the constitutive relationship of concrete refer to the current “Code for Design of Concrete Structures (GB50010-2010) [44]”. The mechanical properties of concrete are shown in Table 4, and Figure 10 provides the damage plasticity (compression damage and tensile damage) data required to establish concrete.

#### 3.2.3. Rebar Parameter Setting in ABAQUS

**Figure 11:**
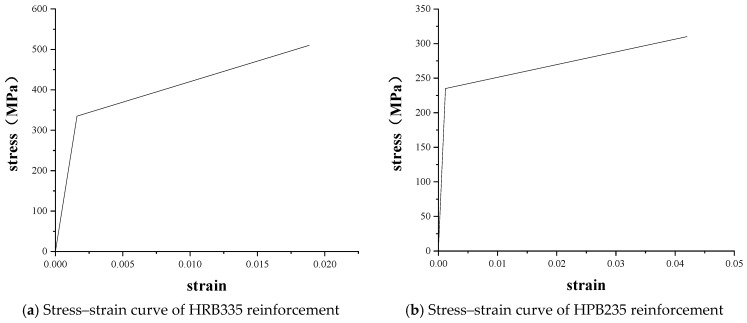
Stress–strain curve of reinforcement.

The longitudinal reinforcement in the slab beam can improve the strength and bearing capacity of the slab beam. The stirrup played the role of shear resistance of the oblique section of the slab beam. At the same time, the longitudinal reinforcement can also be connected as a whole to made it play the role of a skeleton for easy installation. In this paper, the method of defining reinforcement by Shafieifar et al. was used to simulate the longitudinal reinforcement and stirrup by using the elastic-plastic model. The linear elastic behavior of the steel was defined by using the elastic modulus and Poisson’s ratio, and the plastic behavior of the steel was defined by using the yield stress and plastic strain. The mechanical properties and stress–strain curves of the longitudinal reinforcement and stirrup are shown in Table 5 and Figure 11 [45].

#### 3.2.4. Model Establishment and Grid Division

**Figure 12:**
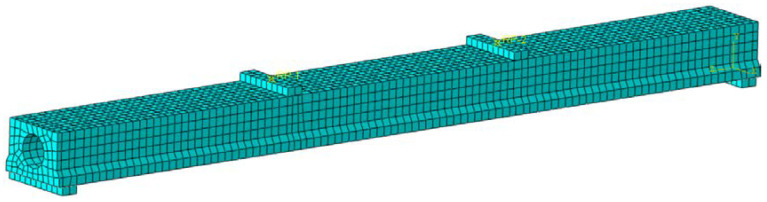
Grid division of the main beam model.

According to the designed member size, the reinforced concrete slab beam model is established by using the separated modeling method. The constitutive relationship between concrete and reinforcement is selected according to Section 3.2.1 and Section 3.2.2 above, and the failure criteria parameters were selected as follows: Dilation Angle = 36, Eccentricity = 0.1, *f*_b0_/*f*_c0_ = 1.16, *K* = 0.667, and Viscosity Parameter = 0.0005. In order to balance the relationship between time cost and calculation accuracy, the concrete element mesh is divided into 25 mm × 25 mm and the grid division size of truss unit: 50 mm × 50 mm. The grid division of the main beam model is shown in Figure 12.

#### 3.2.5. Unit Type

The concrete in the slab beam model selects the solid shrinkage reduction integral element C3D8R, with a total of 5631. The truss unit T3D2 was selected as the reinforcement, with a total of 3229.

#### 3.2.6. Action Mode

In the model built this time, the cushion block and the beam body were connected by binding. The upper surface of the cushion block was coupled to the center point (control point) of the upper surface by means of coupling. The support and beam body were also connected by binding. The reinforcement cage was embedded in the concrete through the built-in area.

#### 3.2.7. Boundary Conditions and Loading Mode

The slab beam model is a test beam, and the support condition at the left end of the beam is set to release only the vertical angular displacement and limit other conditions. The right end releases one more horizontal displacement than the left end, while the other constraints are the same. The load shall be applied step by step.

#### 3.2.8. Verification of the Finite Element Model

**Figure 13:**
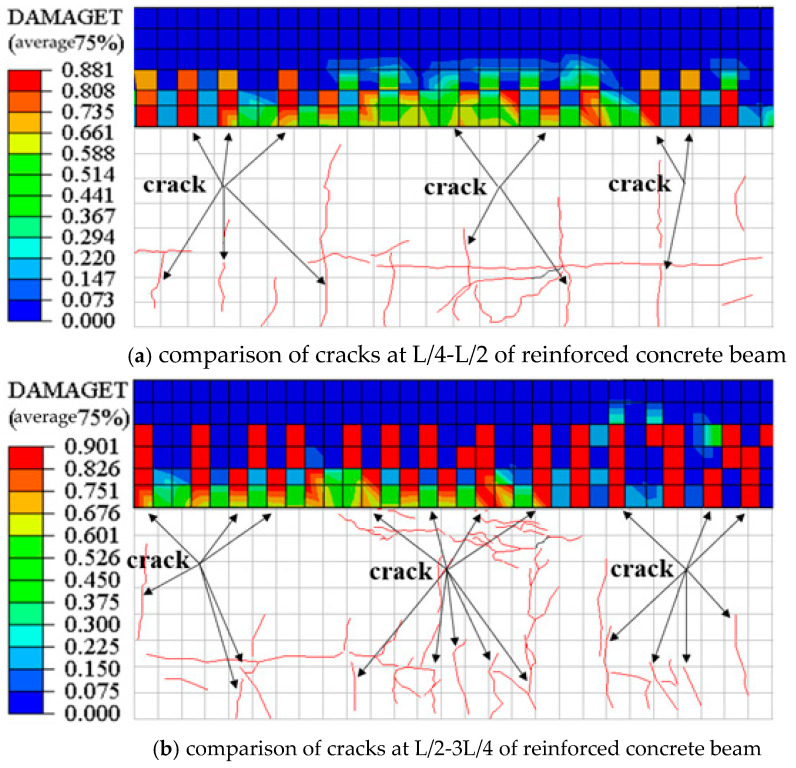
Finite element model crack comparison.

The finite element model can record the failure mode and crack distribution of the beam. When the beam body is under load, the concrete will have vertical compression and lateral expansion inside, resulting in the generation of cracks [46]. In order to ensure the correctness and accuracy of the finite element model, the calculation results of the finite element model were compared with the experimental results. Since the failure mode of the beam is concrete crushing, the crack distribution generated by this failure mode can be effectively recorded by DAMAGET of the finite element model [47]. The specific finite element model crack comparison is shown in Figure 13.

It can be seen from the above figure that the crack distribution of the finite element model under the load is in good agreement with the crack distribution of the main beam after the test, and the DAMAGET values at this time were 0.881 and 0.901, respectively, which were in line with the actual situation. Therefore, the finite element main beam model has high accuracy and accuracy, which can provide accurate basic data for the following work, such as parameter analysis.

### 3.3. Improved MH Sampling

**Figure 14:**
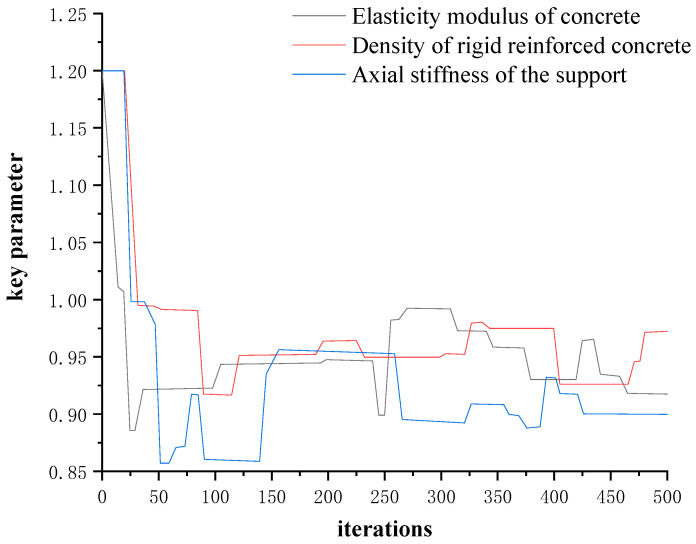
Markov chain convergence results of key parameters of the main beam of the test beam bridge.

According to the analysis results of the sensitivity of key parameters, three key parameters were selected, including E, ρ, and K represents the elastic modulus of concrete, the density of reinforced concrete and the axial stiffness of the bearing. At the same time, the normal random distribution is selected in this paper because it is applicable to random variables that were affected by multiple independent factors, but each influencing factor does not have an advantage (that is, no one factor plays a decisive role, and the influence of each factor is the same), and when these factors were superimposed to affect random variables, then the random variable follows the normal random distribution. The Markov chain starting values of E, ρ, and K were set to 1.2, and the Gaussian normal random distribution is taken as a priori distribution, respectively: E,ρ∈(1.0,0.2), K∈(1.0,0.1). The Markov chain of key parameters is obtained by using the aforementioned procedure for the evaluation of the bearing capacity of test beam bridges based on the Bayesian theory. Finally, the total sample model generated by sampling is 500. We remove the sample models of the first 10% affected by the initial value, and take the remaining 450 sample models to establish a posterior probability distribution library. The Markov chain convergence results of the key parameters of the main beam model of the test beam bridge are shown in Figure 14.

**Figure 15:**
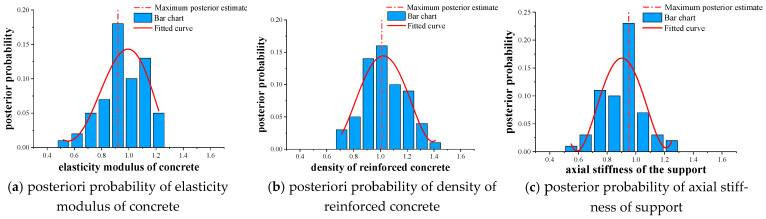
Posterior probability distribution results of the key parameters of the main beam of the test beam bridge.

The posterior probability distribution of each key parameter is shown in Figure 15. The histogram represents the distribution result of the posterior probability, the real rough curve represents the posterior probability after fitting through the generalized extreme curve, and the dotted line represents the maximum posterior estimate. The identification results of the three key parameters based on the improved MH sampling method are shown in Table 6.

### 3.4. TMCMC Sampling

**Figure 16:**
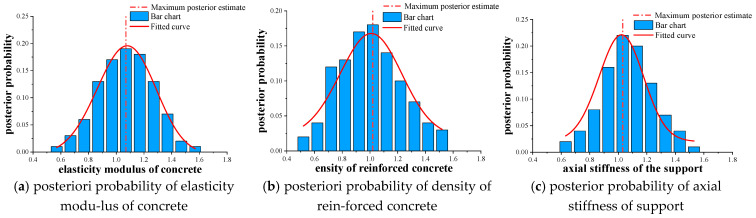
Posterior probability distribution results of the key parameters of the main beam of the test beam bridge.

Figure 14 shows the results of the sensitivity analysis, where the three key parameters of E, ρ, and K take Gaussian normal random distribution as a priori distribution, with N (1.0, 0.2). The TMCMC sampling program was used to generate 500 total sample models, and a posteriori probability distribution library was established. The posterior probability distribution of each key parameter is shown in Figure 16. The histogram represents the distribution result of the posterior probability, the real rough curve represents the posterior probability after normal fitting (Histfit in Matlab), and the dotted line represents the maximum posterior estimate. The identification results of the three key parameters based on the TMCMC sampling method are shown in Table 6.

**Figure 17:**
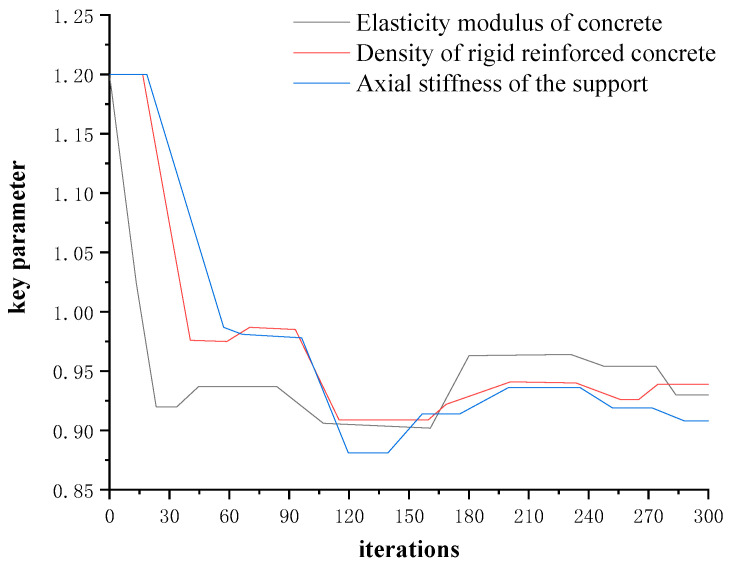
Markov chain convergence results of the key parameters of the main beam of the test beam bridge.

Comparing the results in Figure 15 and Figure 16, it can be seen that the posterior probability distribution results of the key parameters of the main beam obtained by the TMCMC sampling method are better than the improved MH sampling method. Therefore, this paper will explore how to reduce the total sample model generated by sampling to 300, and establish a posterior probability distribution database. Among them, the starting value of Markov chain of E, ρ, and *K* were set to 1.2, and the Gaussian normal random distribution was taken as a priori distribution, respectively: E,ρ∈(1.0,0.2), K∈(1.0,0.1). We removed the sample models of the first 20% affected by the initial value, and used the remaining 240 sample models to establish a posterior probability distribution library. The Markov chain convergence results of the key parameters of the main beam model of the test beam bridge are shown in Figure 17.

According to the analysis results of sensitivity in Figure 17, the three key parameters of E, ρ, and K also take Gaussian normal random distribution as a priori distribution, all of which are *N* (1.0, 0.2). The TMCMC sampling program is used to generate 300 total sample models, and a posteriori probability distribution library is established. The posterior probability distribution of each key parameter is shown in Figure 17. The histogram represents the distribution result of the posterior probability, the real rough curve represents the posterior probability after normal fitting (Histfit in Matlab), and the dotted line represents the maximum posterior estimate. The identification results of the three key parameters based on the TMCMC sampling method are shown in Table 6.

**Figure 18:**
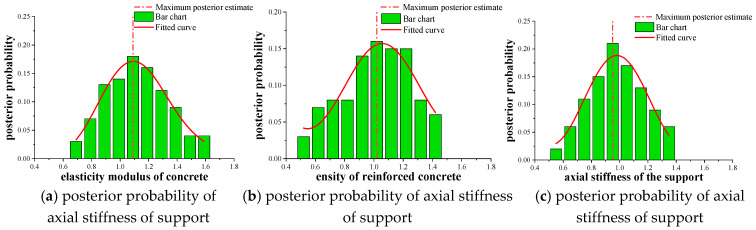
Posterior probability distribution results of the key parameters of the main beam of the test beam bridge.

Comparing Figure 18 with Figure 15, it can be seen that when the total number of models generated by the sampling program is 300, the effect of the recognition results in Figure 18 is not much different from that in Figure 15. However, there is still a big gap between the recognition results in Figure 16, so the number of samples to generate the total model should be controlled at 500, which can better ensure the accuracy of the recognition results.

It can be seen from Table 6 that in the research on the evaluation of the bearing capacity of the test beam bridge based on the Bayesian theory, the identification effect of the improved MH algorithm and TMCMC algorithm is good, which reflects the consistency and progressiveness of the new method for evaluation of bearing capacity of test beam bridge based on the Bayesian theory. This new method is based on the comprehensive analysis of the model error and measurement error of the structure, and uses the probability statistical method to select the model library that best conforms to the real response characteristics of the structure for identification. The identification result is the maximum posterior estimate with statistical law, which further reflects the advantages of this new method.

### 3.5. Prediction and Evaluation of the Bearing Capacity

**Figure 19:**
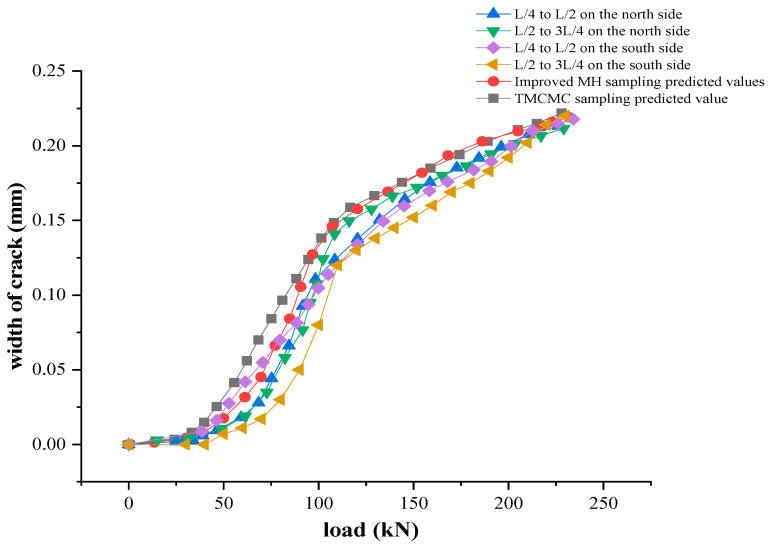
Comparison of the bearing capacity.

Now, the new method of evaluation of the bearing capacity of the test beam bridge based on the Bayesian theory is used to predict the bearing capacity. We substituted the model base data of the above key parameters into the static load calculation of the original model to obtain the bearing capacity prediction results of the main beam. The specific bearing capacity comparison is shown in Figure 19; in order to facilitate the observation of relative error values, the specific values are shown in Table 7.

It can be seen from the figure that the load-crack width prediction curve obtained by improved MH sampling and TMCMC sampling is well matched with the load-crack width test curve obtained by static test. Among them, under the same crack width, the relative error of the bearing capacity obtained from the TMCMC sampling is less than 2.8%, and the relative error of the bearing capacity obtained from the improved MH sampling is less than 3.3%, and the measured value of the bearing capacity is within the confidence interval. This result reflects the feasibility and effectiveness of the new method for the evaluation of the bearing capacity of the test beam bridge based on the Bayesian theory.

## 4. Conclusions

(1) Based on the Bayesian theory, the new method for evaluation of the bearing capacity of test beam bridges was to comprehensively analyze the cognitive error and random error of the structure, combine each “model components” into a model sample, and select the model library most suitable for the structural performance characteristics from the complex model samples by comparing the test results and the prediction results of the finite element model. Then, the probability distribution of the structural response prediction was obtained by using the model base and the performance evaluation was completed. The results are of statistical significance and more scientific and accurate.
(2) This paper introduced the traditional Markov Chain-Monte Carlo Simulation (MCMC) sampling method, and then proposes two optimized MCMC sampling methods: an improved MH sampling method and a TMCMC sampling method. It can overcome the problems of traditional MCMC method, such as difficult convergence and low computational efficiency, when the model is complex and the parameter dimension is high. The derived MCMC sampling method improved by the above optimization can better achieve the evaluation of the bearing capacity of the structure.
(3) Based on the static test results of reinforced concrete beams, the finite element model of the reinforced concrete beam was established by selecting three model components. The elastic modulus of concrete, the density of reinforced concrete and the axial stiffness of the support, and the sensitivity analysis of the model components was carried out. Based on the Bayesian theory, a posteriori probability distribution library was established using ABAQUS finite element software and Matlab. The improved MH sampling method and TMCMC sampling method were used to obtain the load-crack width curve of the reinforced concrete beam. The comparison shows that the prediction curve and the experimental test curve fit well. Among them, under the same crack width, the relative error of the bearing capacity obtained from the TMCMC sampling was less than 2.8%, and the relative error of the bearing capacity obtained from the improved MH sampling was less than 3.3%. Moreover, the measured value of the bearing capacity was within the confidence interval, which reflected the feasibility and effectiveness of the new method for the evaluation of the bearing capacity of the test beam bridge based on the Bayesian theory.

## Figures and Tables

**Table 1:** Loading classification of the main beam.

Classification	Loading Value (kN)	Classification	Loading Value (kN)	Classification	Loading Value (kN)
preload level 1	5	level 11	95	level 25	235
preload level 2	10	level 12	105	level 26	245
preload level 3	15	level 13	115	level 27	255
uninstall	0	level 14	125	level 28	265
level 1	5	level 15	135	level 29	275
level 2	10	level 16	145	level 30	285
level 3	15	level 17	155	level 31	295
level 4	25	level 18	165	level 32	305
level 5	35	level 19	175	level 33	315
level 6	45	level 20	185	level 34	350
level 7	55	level 21	195	level 35	365
level 8	65	level 22	205	level 36	380
level 9	75	level 23	215	level 37	395
level 10	85	level 24	225	level 38	410

Note: The preload is unloaded by stages, and the final loading value is determined by the failure characteristics of the test beam.

**Table 2:** Parameter values of the sensitivity analysis.

Model Components	Parameter Lower Limit	Parameter Upper Limit	Number of Samples	Proportional Spacing
elastic modulus *E*	0.9 *E*_0_	1.5 *E*_0_	15	linear average
Density *ρ*	0.6 *ρ*_0_	1.2 *ρ*_0_	15	linear average
support stiffness *K*	0.01 *K*_0_	100 *K*_0_	15	logarithmic average

Note: *E*_0_ is the elastic modulus of concrete, *ρ*_0_ is the density of reinforced concrete, and *K*_0_ is the axial stiffness of the bearing.

**Table 3:** Damage plastic parameters.

Parameter	Reference Value
*ψ*	36°
*e*	0.1
*f*_b0_/*f*_c0_	1.16
*K*	0.667
*μ*	0.0005

**Table 4:** Mechanical properties of C40 concrete.

Parameter	*E_0_* (MPa)	*v*	*f′_c_* (MPa)	*f_t_* (MPa)
**Reference Value**	32,500	0.2	26.8	2.4

**Table 5:** Mechanical properties of reinforcement.

Parameter	*E* (MPa)	ν	Yield Stress (MPa)	Yield Strain	Ultimate Strength (MPa)	Ultimate Strain
HRB335	210,000	0.3	335	0.00160	510	0.01884
HPB235	210,000	0.3	235	0.00120	310	0.04200

**Table 6:** Comparison of the identification results of the key parameters of the main beam of the test beam bridge.

Standardized Key Parameters	Improved MH Algorithm (500 Sample Models)	TMCMC Algorithm (500 Sample Models)	TMCMC Algorithm (300 Sample Models)
*E*	0.97	1.06	1.09
*ρ*	1.03	0.98	1.02
*K*	0.91	1.02	0.93

**Table 7:** Calculation of the relative error of the bearing capacity.

Crack Width (mm)	Test Measured Value (kN)	Predicted Value of Improved MH Algorithm (kN)	Relative Error	Predicted Value of TMCMC Algorithm (kN)	Relative Error
0.13	100.1	96.9	3.2%	97.8	2.3%
0.14	108.6	105.4	3.0%	106.1	2.3%
0.15	120.0	116.1	3.3%	118.4	1.4%
0.16	127.4	125.3	1.7%	124.6	2.2%
0.17	138.2	135.7	1.8%	134.4	2.8%
0.18	151.2	146.9	2.9%	147.7	2.4%
0.19	169.8	167.7	1.3%	166.3	2.1%
0.20	192.3	188.8	1.9%	190.3	1.1%
0.21	211.7	206.5	2.5%	208.2	1.7%
0.22	232.0	228.2	1.7%	229.1	1.3%

Note: relative error = (measured value − predicted value)/measured value.

## Data Availability

The data presented in this study are available on request from the corresponding author. The data are not publicly available due to privacy.

## Nomenclature

*φ*	diameter of reinforcement
HRB	hot rolled ribbed steel bar
HPB	hot rolled plain round steel bar
C40	concrete with compressive strength of 40 MPa
*Fu*	calculated bearing capacity
*Fr*	flexural tensile strength of concrete
*E*	elastic modulus of concrete
*ρ*	reinforced concrete density
*K*	axial stiffness of support
*Ψ*	expansion angle
*e*	eccentricity
*f_b0_*/*f_c0_*	ultimate strength ratio of biaxial bearing and uniaxial bearing
*μ*	coefficient of viscosity
*v*	Poisson ratio
*f′c*	axial compressive strength of concrete
*f_t_*	tensile strength of concrete
